# Interleukin-25 is detrimental for recovery after spinal cord injury in mice

**DOI:** 10.1186/s12974-016-0566-y

**Published:** 2016-05-06

**Authors:** Dearbhaile Dooley, Evi Lemmens, Peter Ponsaerts, Sven Hendrix

**Affiliations:** Department of Morphology, Biomedical Research Institute, Hasselt University, Diepenbeek, Belgium; Laboratory of Experimental Hematology, Vaccine and Infectious Disease Institute (Vaxinfectio), University of Antwerp, Antwerp, Belgium

**Keywords:** IL-25, Type 2 immune response, Spinal cord injury, Basso mouse scale, Locomotor recovery, Th1, Th2

## Abstract

**Background:**

The cytokine, interleukin (IL)-25, is thought to be critically involved in inducing a type 2 immune response which may contribute to regeneration after central nervous system (CNS) trauma. We investigated whether applying recombinant IL-25, locally or systemically, in a mouse model of spinal cord injury (SCI) improves functional and histological recovery.

**Findings:**

Repeated systemic administration of IL-25 did not influence functional recovery following SCI. In contrast, a single local administration of IL-25 significantly worsened locomotor outcome, which was evident from a decreased Basso mouse scale (BMS) score compared with phosphate-buffered saline (PBS)-treated controls. This was accompanied by a significant increase in lesion size, demyelination, and T helper cell infiltration.

**Conclusions:**

These data show for the first time that IL-25 is either ineffective when applied systemically or detrimental to spinal cord recovery when applied locally. Our findings question the potential *neuroprotective* role of IL-25 following CNS trauma.

**Electronic supplementary material:**

The online version of this article (doi:10.1186/s12974-016-0566-y) contains supplementary material, which is available to authorized users.

## Findings

### Introduction

For decades, it has been the general opinion that an imbalanced immune response plays a major role in the pathophysiology of central nervous system (CNS) trauma and disease. Inflammation may prove problematic for many repair processes [1] but may also exert beneficial effects when closely regulated. The type 2 response can be characterized by differentiation of CD4^+^ T helper type 2 (Th2) cells and the production of the type 2 cytokines interleukin-4 (IL-4), IL-5, IL-9, and IL-13 [2–4]. This in turn inhibits phagocytosis [5] and suppresses inflammatory cytokines [6]. Therefore, type 2 immune factors can contribute to immune regulation by suppressing excessive pro-inflammatory processes [7, 8].

We as well as others have shown that cytokines associated with Th2 cells such as IL-4 [9–11] and IL-10 [12, 13], not only promote neuronal survival and regeneration but also improve functional outcome after CNS trauma such as SCI. IL-25 (also known as IL-17E) has been suggested to be a key player in the origin of a type 2 response [2, 14]. While research has begun to unravel its importance in immunity in general, conclusive data on the role of IL-25 in the CNS is lacking. Although a limited number of studies are currently available, these tend to point towards a *protective* role of IL-25 in neuroinflammation (reviewed in [15, 16]). For example, IL-25 treatment suppresses Th17 responses and disease symptoms in experimental autoimmune encephalomyelitis (EAE) and is important in maintaining blood-brain barrier function [17, 18]. IL-25 expression is downregulated by pro-inflammatory cytokines such as tumor necrosis factor-α and IL-1β, which increase acutely after trauma. Consistently, IL-25 is reduced in the pro-inflammatory milieu of CNS lesions [17]. These findings suggest that an increase in IL-25 may possess the therapeutic potential to provide repair after CNS trauma.

In the present short report, we tested whether recombinant murine IL-25, administered either as a single dose locally to the spinal cord or via repeated systemic injections, improves functional recovery after SCI in mice. While no clinical effect was observed following systemic administration of IL-25, surprisingly, when applied locally, IL-25 lead to a significant decrease in locomotor recovery as well as a substantial increase in lesion size, demyelination, and T helper cell infiltration.

### Methods

A T-cut hemisection injury was performed as previously described [19–22] in 10-week-old female BALB/c mice (Harlan, The Netherlands). See supplementary materials for details. Mice were treated with recombinant murine IL-25 (500 ng or 1 μg; ImmunoTools, Germany) via two different methods. Mice received either a single, local application of IL-25 (1 μg), by placing a cytokine-saturated gelfoam patch at the lesion site immediately after injury, or systemic administration via repeated intraperitoneal (i.p.) injections (500 ng) once daily for 7 days starting 1 day before injury. The dose for the local application of IL-25 was chosen based on pilot experiments in our lab, where we observed a non-significant trend towards a decreased functional recovery after SCI following treatment with a lower dose (500 ng/ml; data not shown). The dose for systemic administration was chosen based on a previous study [17]. Control animals were treated with vehicle, i.e., phosphate-buffered saline (PBS) (*n* = 7–10 mice/group). All experiments were performed according to the guidelines of EU Directive 2010/63/EU on the protection of animals used for scientific purposes and were approved by the local ethical committee for animal experimentation at Hasselt University.

Starting 1 day after surgery, functional recovery in SCI mice was measured at regular time points for 3 weeks using the Basso mouse scale (BMS) [23] as previously described [19, 20, 22]. Histological analysis was performed on mice receiving a local and systemic application of IL-25 as previously described [20, 22]. The following antibodies were analyzed: anti-glial fibrillary acidic protein (GFAP; Sigma-Aldrich, Belgium), anti-myelin basic protein (MBP; Millipore, Belgium), anti-CD4 (BD biosciences, Belgium), and anti-ionized calcium-binding adaptor molecule 1 (Iba-1; Wako, Germany). See Additional file 1 for details.

For quantification of astrogliosis (GFAP) and microglia/macrophage infiltration (Iba-1), TissueQuest immunofluorescence analysis software (TissueGnostics GmbH, v3.0) was used, as previously described [24]. Pictures were taken using a Nikon Eclipse 80i microscope (Nikon, Brussels, Belgium). See Additional file 1 for details.

To study the effect of IL-25 on cell survival in vitro, we used a human astrocytoma cell line (CCF) [25], a human glial (oligodendrocytic) hybrid cell line (MO3.13) [26], an immortalized murine BV-2 cell line [27], and primary cortical neuronal cells as previously described [3]. All cell types were grown under optimal conditions and treated with selected concentrations of IL-25 (5 ng/ml, 50 ng/ml, 500 ng/ml, and 1 μg/ml) for 72 h to measure viability. See Additional file 1 for details.

Statistical analyses were performed using GraphPad Prism software (GraphPad Software Inc., USA). Differences between treatment groups in lesion size, demyelinated area, and T cell numbers were calculated using the Mann-Whitney *U* test. Differences in astrogliosis and microglia/macrophage infiltration, as well as in the BMS data, were analyzed using the two-way ANOVA for repeated measurements (with Bonferroni post hoc tests). Differences were considered to be significant when *p* < 0.05. Data in graphs are presented as mean ± SEM.

### Results and discussion

**Fig. 1:**
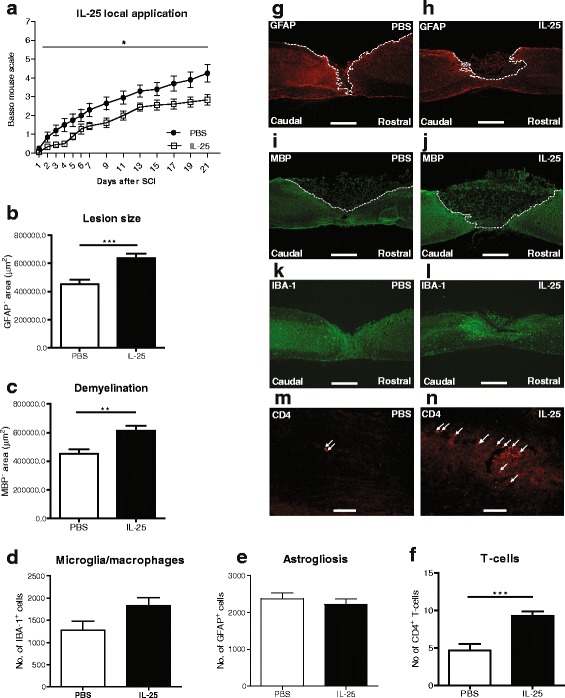
Local application of IL-25 decreases functional outcome and increases lesion size, demyelination, and T cell infiltration following SCI in mice. (**a**) Mice receiving local application of IL-25 show a statistically significant decrease in functional outcome when compared to those receiving PBS, as measured by the BMS (*p < 0.05), *n* = 9–10 mice/group. (**b**) Lesion size and (**c**) demyelinated area were quantified by staining for (**g**, **h**) GFAP and (**i**, **j**) MBP, respectively, as depicted by the *dotted white line*. Image analysis revealed a significant increase in (**b**) lesion size and (**c**) demyelinated area in animals treated locally with IL-25, compared with the PBS control group. Quantification of (**d**) Iba-1^+^ and (**e**) GFAP^+^ cells after SCI using TissueQuest software revealed no significant difference in (**k**, **l**) microglia/macrophages numbers or (**g**, **h** astrogliosis between animals receiving PBS or IL-25. (**f**) Significantly more CD4^+^ T cells are present in the spinal cord sections of the (**n**) IL-25-treated mice, compared with (**m**) PBS-treated mice, 3 weeks after SCI. *Scale bars* of representative photomicrographs: (**g**–**l**) = 500 μm, **m** + **n** = 50 μm. Data represent mean ± SEM. ***p < 0.001, **p < 0.01, *n* = 5–6 mice/group

**Fig. 2:**
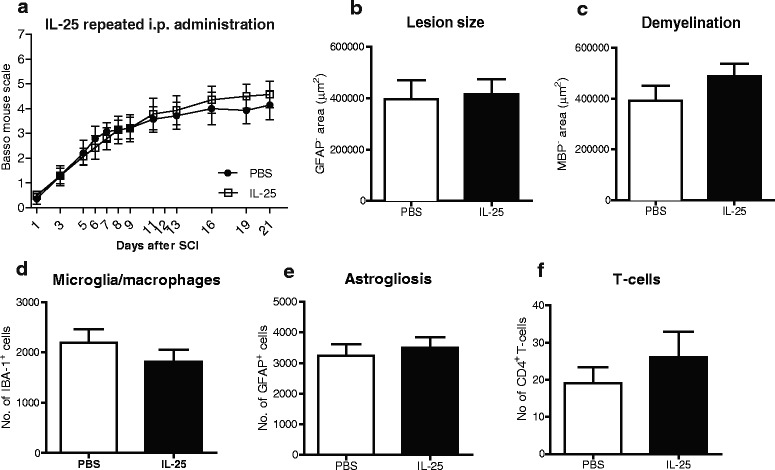
Systemic application of IL-25 has no effect on functional or histological outcome following SCI in mice. (**a**) Repeated i.p. administration of IL-25 has no significant effect on functional outcome when compared to those receiving PBS, as measured by the BMS, *n* = 7 mice/group. Sections were stained for GFAP and MBP to determine the (**b**) lesion size and (**c**) demyelinated area, respectively. Image analysis revealed no significant difference in (**b**) lesion size or (**c**) demyelinated area in animals treated systemically with IL-25, compared with the PBS control group. Quantification of (**d**) Iba-1^+^ and (**e**) GFAP^+^ cells after SCI using TissueQuest software revealed no significant difference in microglia/macrophages numbers or astrogliosis between animals receiving PBS or IL-25. (**f**) There was no significant difference in the number of CD4^+^ T cells present in spinal cord sections of IL-25-treated mice, compared with PBS-treated mice. Data represent mean ± SEM, *n* = 7 mice/group

In this short report, we investigated whether increasing levels of IL-25, a potential inducer of a type 2 immune response, can promote functional recovery in a mouse model of SCI. Considering the widespread expression of the receptor A subunit of the IL-17 receptor which forms a complex with the receptor B subunit upon binding with IL-25 [28], we aimed to distinguish between local and systemic effects of treatment. We found that local application of IL-25 led to a significant worsening in motor performance following injury compared with PBS controls (Fig. 1a; *p < 0.05). At the histological level, these results were accompanied by a 30 % increase in lesion size (Fig. 1b, g, h; ***p < 0.001) and demyelinated area (Fig. 1c, i, j; **p < 0.01). Surprisingly, systemic IL-25 treatment did not influence functional recovery (Fig. 2a). Furthermore, there was no effect of systemic IL-25 treatment on lesion size or demyelinated area (Fig. 2b, c).

We also analyzed the presence of microglia/macrophages (Figs. 1d, k, l and 2d) as well as astrogliosis (Figs. 1e, g, h and 2e), by quantifying the number of Iba-1+ and GFAP+ cells, respectively. However, no significant differences were found between IL-25 treated and control groups, in both local and systemic treatment. Following quantification of perilesional CD4^+^ T cells, we found a significant increase in the number of cells in tissue sections from mice treated locally with IL-25, compared with PBS controls (Fig. 1f, m, n; ***p < 0.001). There was no effect of systemic IL-25 treatment on the number of CD4^+^ T cells (Fig. 2f). The precise role of T cells following CNS injury is still subject to discussion. Although they may display beneficial effects under certain conditions, accumulation of endogenous T cells may be considered detrimental [7, 20].

We also investigated the effect of IL-25 in vitro on cell viability. However, we observed no significant effects of various concentrations of IL-25 on survival of oligodendrocytes, astrocytes, microglia, or primary cortical neuronal cells (Additional file 2: Figure S1A-D). These results may be consistent with the lacking effect of systemic IL-25 treatment in vivo. They also indicate that the toxic effect observed locally is not caused by a direct effect on the above cell types. This suggests that local administration of IL-25 following SCI activates an indirectly mediated cascade of detrimental immune events.

Although a member of the rather *pro-inflammatory* IL-17 family, IL-25 plays a somewhat different role in the context of CNS inflammation. IL-25 messenger RNA (mRNA) is highly expressed in polarized Th2 cells [14], and IL-25 administration in mice drives the Th2 response, by elevating IL-4 and IL-13 levels [14, 16]. Systemic IL-25 regulates the development of autoimmune inflammation mediated by IL-17-producing cells and suppresses EAE symptoms in a relapse-remitting model [14]. Additionally, the delivery of IL-25 to the CNS in two different models of neuroinflammation was able to drive microglia and macrophages to a more anti-inflammatory and tissue-protective phenotype [29].

In contrast to the above positive effects on neuroinflammation, our results indicate that systemic administration of IL-25 after SCI in mice is ineffective in improving functional outcome. This result was surprising given that we as well as others have shown that treatment with cytokines which induce a type 2 response, such as IL-4 and IL-10, are neuroprotective following SCI [11, 12]. Differences in systemic versus local administration is a well-known phenomenon [30–32], and our results are consistent with this as local application of IL-25 decreased functional recovery after SCI. Furthermore, we observed that a lower local dose of IL-25 (500 ng/ml) leads to a non-significant trend towards a decrease in functional outcome after SCI (data not shown), indicating that route of administration and dosing are important factors which must be considered prior to use of cytokine therapy.

Interestingly, it was previously demonstrated that intraspinal treatment with IL-10 exacerbated damage and lesion size, while when given systemically, it improved recovery after SCI [12]. Taken together, these data reiterate the well-recognized fact that the route of administration is of pivotal importance when determining a therapeutic outcome. Additionally, Mearns et al. recently questioned the role of IL-25 in Th2 cell differentiation and the induction of potentially beneficial Th2-cell responses [33]. In contrast to previous reports, the authors used reporter mouse technology to show that IL-25 is dispensable during differentiation and development of Th2 cells [33]. In our study, IL-25 failed to have an effect systemically and even worsened functional outcome when applied locally. This suggests that the direct involvement of IL-25 in driving a Th2 response remains questionable. Furthermore, based on the current opinion on the role of Th2 cytokines following CNS injury [8], it is safe to suggest that factors which regulate the type 2 immune response are, in turn, key players in CNS pathology.

In this short report, we show for the first time that IL-25 is either ineffective when applied systemically or detrimental to spinal cord recovery when applied locally. These findings indicate that the potential positive effects of IL-25 and its involvement in driving a beneficial type 2 immune response need to be carefully reconsidered prior to its use therapeutically.

## Additional files

Additional file 1: Supplementary Materials. Detailed description of materials and methods used throghout the manuscript, provided as supplementary information. (DOCX 35 kb)

Additional file 2: Figure S1. IL-25 has no effect on mature oligodendrocyte, astrocyte, microglia, or primary neuron cell viability. **(A)** MO3.13 cells were differentiated to mature oligodendrocytes using PMA for 72 h and were treated for 48 h with selected concentrations of IL-25. **(B, C)** The astrocytic and microglial cell lines (CCF and BV2, respectively) were treated for 48 h with selected concentrations of IL-25. **(D)** Primary neurons were incubated with selected concentrations of IL-25 for 48 h in the presence or absence of B27. B27 deprivation induced a decreased cell viability, but IL-25 treatment had no effect on this. The selected concentrations of IL-25 used for all cell types were 5 ng/ml, 50 ng/ml, 500 ng/ml, and 1 μg/ml. Cell survival was measured using an MTT assay, and values are expressed as percentage of the control. **(A-D)** There was no significant effect observed on cell viability in all cell types tested. Data represent mean ± SEM of one representative experiment (from two to three independent experiments) ***p < 0.001. (PDF 316 kb)

## Abbreviations

BMS: Basso mouse scale
CNS: central nervous system
GFAP: glial fibrillary acidic protein
i.p.: intraperitoneal
Iba-1: ionized calcium-binding adaptor molecule 1
IL: interleukin
MBP: myelin basic protein
PBS: phosphate-buffered saline
SCI: spinal cord injury
