# Supplementary material for: Interleukin-25 is detrimental for recovery after spinal cord injury in mice
Source: J Neuroinflammation. 2016 May 6;13:101. doi: 10.1186/s12974-016-0566-y (PMC4858907; doi:10.1186/s12974-016-0566-y)
Supplement: Supplementary file 1 — Supplementary Materials. Detailed description of materials and methods used throghout the manuscript, provided as supplementary information. (DOCX 35 kb) [file 12974_2016_566_MOESM1_ESM.docx]

**Methods**

*Spinal cord T-cut hemisection injury*

A T-cut hemisection injury was performed as previously described [[1-4](#_ENREF_1)] in 10-week-old female BALB/c mice (Harlan, The Netherlands). In brief, 10-week-old female BALB/c mice were anesthetized to undergo a partial laminectomy at thoracic level 8. Next, iridectomy scissors were used to transect left and right posterior columns, the dorsal horns and additionally the ventral funiculus. Finally, the muscles were sutured and the back skin closed with wound clips. It is important to mention is that this procedure (i.e. T-cut) results in a complete transection of the corticospinal tract and impairment of several other descending and ascending motor and sensory tracts. All experiments were performed according to the guidelines of EU Directive 2010/63/EU on the protection of animals used for scientific purposes and were approved by the local ethical committee for animal experimentation at Hasselt University.

*Locomotion tests*

Starting 1 day after surgery, functional recovery in SCI mice was measured at regular time points for three weeks using the BMS [[5](#_ENREF_5)]. The BMS is a 10-point locomotor rating scale (9 = normal locomotion; 0 = complete hind limb paralysis), in which mice are scored by two investigators blinded to the experimental groups. The given scores are based on hind limb movements made in an open field during a 4-minute interval.

*Immunofluorescence protocol*

Three weeks after surgery, mice were anesthetized and transcardially perfused with Ringer’s solution containing heparin, followed by perfusion with 4% paraformaldehyde. Spinal cord tissue was resected and cryoprotected in sucrose (5% followed by 30%), then stored at -80°C prior to sectioning. To analyze lesion size, demyelination, astrogliosis as well as T cell and microglia/macrophage infiltration, cryosections (10 µm) were pretreated with PBS (pH 7.4) containing 10% normal goat serum and 0.05% Triton X-100 for 1h. Sections were then incubated for 2h at room temperature or overnight at 4°C with the following primary antibodies, diluted in PBS containing 1% normal goat serum and 0.05% Triton X-100: a combination of mouse anti-glial fibrillary acidic protein (GFAP) (1:500; Sigma-Aldrich, Belgium) and rabbit anti-myelin basic protein (MBP) (1:500; Millipore, Belgium), or with rat anti-CD4 (1:500; BD biosciences, Belgium) and rabbit anti-ionized calcium binding adaptor molecule 1 (Iba-1) (1:350; Wako, Germany). Subsequently, sections were washed with PBS, and corresponding secondary antibodies were applied for 1h at room temperature: goat anti-mouse Alexa Fluor 568, goat anti-rabbit Alexa Fluor 488, or goat anti-rat Alexa Fluor 568 (dilution 1:250 in PBS containing 1% normal goat serum and 0.05% Triton X-100; Life Technologies, Belgium). Finally, a DAPI nuclear stain was performed before sections were mounted using anti-fade fluorescent mounting medium (Dako, Germany). Negative controls were prepared by omitting incubation with the primary antibody from the protocol.

*Image analysis*

Image analysis was performed using pictures taken by a Nikon Eclipse 80i microscope (Nikon, Brussels, Belgium), with one series containing a maximum of 8 sections per animal per analysis, as previously described [[1](#_ENREF_1), [2](#_ENREF_2)], with minor modifications. Lesion size and demyelinated area were defined by delineating the area devoid of GFAP or MBP immunoreactivity, respectively. To quantify the number of infiltrating CD4^+^ T helper cells, the entire section containing the lesion epicenter as well as the perilesional area was used. CD4-positive but Iba-1 negative cells (to exclude CD4^+^ microglia/macrophages) were counted manually. For quantification of astrogliosis (GFAP) and microglia/macrophage infiltration (Iba-1), TissueQuest immunofluorescence analysis software (TissueGnostics GmbH, v3.0) was used, as previously described [[6](#_ENREF_6)]. Each slide was analysed at 4X magnification and the number of IBA-1+ and GFAP+ cells at the lesion site and surrounding tissue were quantified based on a DAPI nuclear staining.

*Cell cytotoxicity assay*

Primary cortical neuronal cells were isolated from embryonic day 15 (E15) BALB/c mice and were cultured in neuronal media (Neurobasal media, containing 2% B27, 1% glutamine and 1% penicillin/streptomycin). MO3.13, CCF and BV2 cell lines were used to study survival of oligodendrocytes, astrocytes and microglia respectively. All cells were seeded on poly-d-lysine-coated 96-wells at a density of 5 x 10^3^ cells/well. Cells were grown under optimal conditions and treated with selected concentrations of IL-25 (5 ng/ml, 50 ng/ml, 500 ng/ml, and 1 μg/ml for 72 hours to measure viability. To measure a potential beneficial effect of IL-25 inhibition on neuronal death, 1 hour after isolation, cells were incubated in B27 deficient media with or without IL-25 for 72 hours. CCF cells were cultured in DMEM/F-12 medium containing 10% FCS and 1% penicillin/streptomycin and BV2 cells were cultured in (DMEM medium, containing 10% FCS and 1% penicillin/streptomycin. MO3.13 cells were cultured in MEM medium with 10% FCS and 1% penicillin/streptomycin. After 70% confluency, MO3.13 cells were differentiated in DMEM medium by removing FCS from the culture medium and adding 100nM 4-α-phorbol 12-myristate 13-acetate (PMA) to the cells for 72 hours [[7](#_ENREF_7), [8](#_ENREF_8)]. Following treatment of all cell types with varying concentrations of IL-25, a 3-(4,5-dimethylthiazol-2-yl)-2,5-diphenyl tetrazolium bromide (MTT) solution (1 mg/ml) was added for 4 h. The cells were lysed in a mixture of dimethyl sulfoxide (DMSO) and glycine (0.1 M), and the absorption was measured at 540nm using a microplate reader (Bio-Rad, Nazareth, Belgium). Cells treated with 10% DMSO were used as positive control.

**Supplementary References**

1. Boato F, Hendrix S, Huelsenbeck SC, Hofmann F, Grosse G, Djalali S et al. C3 peptide enhances recovery from spinal cord injury by improved regenerative growth of descending fiber tracts. Journal of cell science. 2010;123(Pt 10):1652-62. doi:10.1242/jcs.066050.

2. Nelissen S, Vangansewinkel T, Geurts N, Geboes L, Lemmens E, Vidal PM et al. Mast cells protect from post-traumatic spinal cord damage in mice by degrading inflammation-associated cytokines via mouse mast cell protease 4. Neurobiology of disease. 2014;62:260-72. doi:10.1016/j.nbd.2013.09.012.

3. Tuszynski MH, Steward O. Concepts and methods for the study of axonal regeneration in the CNS. Neuron. 2012;74(5):777-91. doi:10.1016/j.neuron.2012.05.006.

4. Geurts N, Vangansewinkel T, Lemmens S, Nelissen S, Geboes L, Schwartz C et al. Basophils are dispensable for the recovery of gross locomotion after spinal cord hemisection injury. Journal of Leukocyte Biology. 2015. doi:10.1189/jlb.3AB0815-370R.

5. Basso DM, Fisher LC, Anderson AJ, Jakeman LB, McTigue DM, Popovich PG. Basso Mouse Scale for locomotion detects differences in recovery after spinal cord injury in five common mouse strains. Journal of neurotrauma. 2006;23(5):635-59. doi:10.1089/neu.2006.23.635.

6. Le Blon D, Hoornaert C, Daans J, Santermans E, Hens N, Goossens H et al. Distinct spatial distribution of microglia and macrophages following mesenchymal stem cell implantation in mouse brain. Immunol Cell Biol. 2014;92(8):650-8. doi:10.1038/icb.2014.49.

7. Kim WH, Lee JW, Gao B, Jung MH. Synergistic activation of JNK/SAPK induced by TNF-α and IFN-γ: Apoptosis of pancreatic β-cells via the p53 and ROS pathway. Cellular Signalling. 2005;17(12):1516-32. doi:<http://dx.doi.org/10.1016/j.cellsig.2005.03.020>.

8. Vidal PM, Lemmens E, Avila A, Vangansewinkel T, Chalaris A, Rose-John S et al. ADAM17 is a survival factor for microglial cells in vitro and in vivo after spinal cord injury in mice. Cell Death & Disease. 2013;4(12):e954. doi:10.1038/cddis.2013.466.
