# Supplementary figures and images for: Interleukin-25 is detrimental for recovery after spinal cord injury in mice
Source: J Neuroinflammation. 2016 May 6;13:101. doi: 10.1186/s12974-016-0566-y (PMC4858907; doi:10.1186/s12974-016-0566-y)

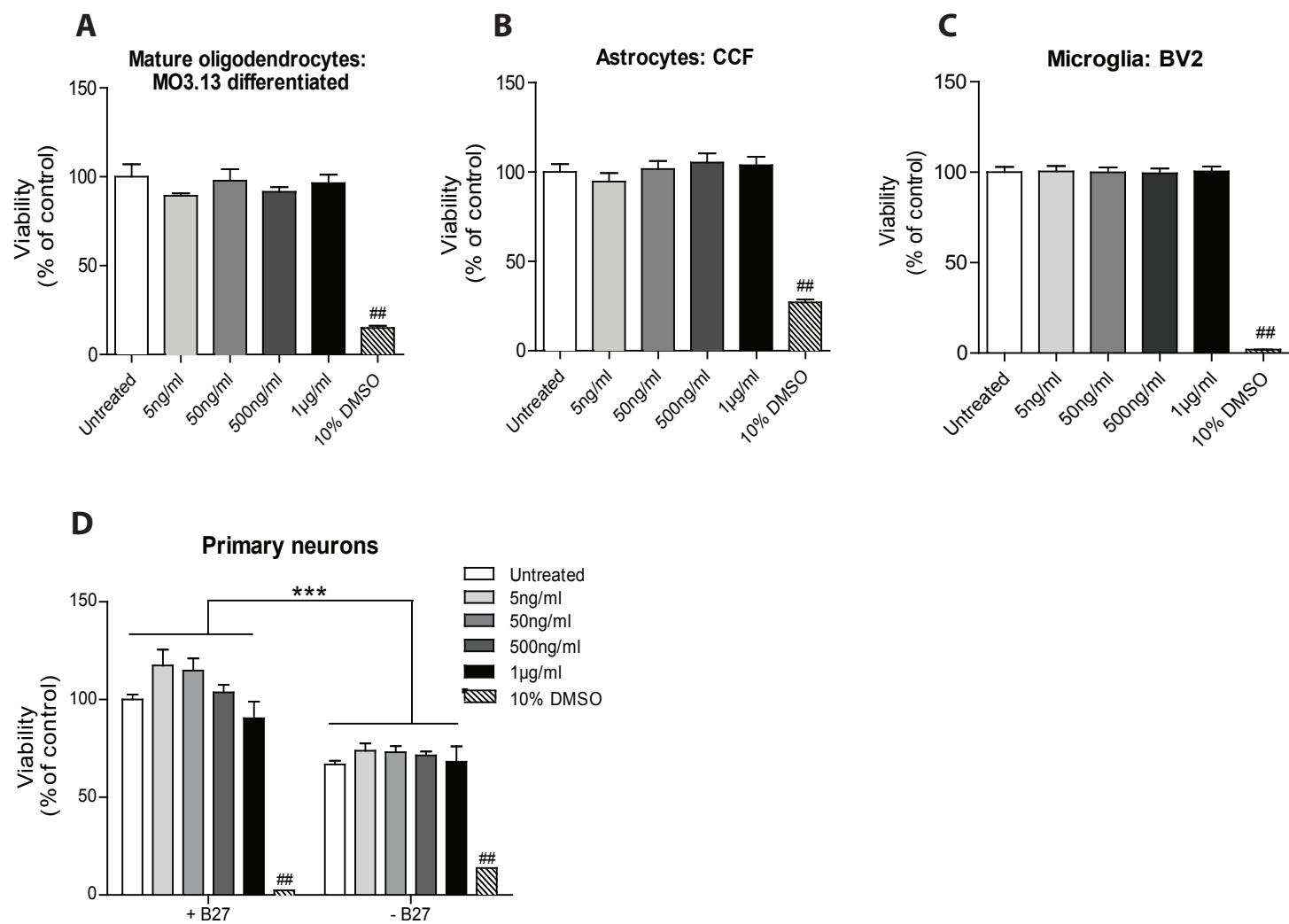

Supplement: Supplementary file 2 — IL-25 has no effect on mature oligodendrocyte, astrocyte, microglia, or primary neuron cell viability. (A) MO3.13 cells were differentiated to mature oligodendrocytes using PMA for 72 h and were treated for 48 h with selected concentrations of IL-25. (B, C) The astrocytic and microglial cell lines (CCF and BV2, respectively) were treated for 48 h with selected concentrations of IL-25. (D) Primary neurons were incubated with selected concentrations of IL-25 for 48 h in the presence or absence of B27. B27 deprivation induced a decreased cell viability, but IL-25 treatment had no effect on this. The selected concentrations of IL-25 used for all cell types were 5 ng/ml, 50 ng/ml, 500 ng/ml, and 1 μg/ml. Cell survival was measured using an MTT assay, and values are expressed as percentage of the control. (A-D) There was no significant effect observed on cell viability in all cell types tested. Data represent mean ± SEM of one representative experiment (from two to three independent experiments) ***p < 0.001. (PDF 316 kb) [file 12974_2016_566_MOESM2_ESM.pdf]
